# Immune checkpoint inhibitor infusion times and clinical outcomes in patients with melanoma

**DOI:** 10.1093/oncolo/oyae197

**Published:** 2024-08-27

**Authors:** Kylie Fletcher, Saba Rehman, Rebecca Irlmeier, Fei Ye, Douglas Johnson

**Affiliations:** Vanderbilt University School of Medicine, Nashville, TN, United States; Vanderbilt University School of Medicine, Nashville, TN, United States; Department of Biostatistics, Vanderbilt University Medical Center, Nashville, TN, United States; Department of Biostatistics, Vanderbilt University Medical Center, Nashville, TN, United States; Department of Hematology/Oncology, Vanderbilt University Medical Center, Nashville, TN, United States

**Keywords:** melanoma, immunotherapy, circadian rhythm, immune checkpoint inhibitor

## Abstract

**Background:**

Circadian rhythms impact immune function; a previous study demonstrated that immunotherapy treatment times taking place later in the day correlated with poorer outcomes in patients with melanoma. However, this finding has not been replicated, and other infusion timing schemas are unexplored. The objective of this retrospective, cohort study was to determine if the time of immunotherapy infusion affects outcomes.

**Materials and Methods:**

Five hundred and sixteen participants age ≥18 years diagnosed with cutaneous, acral, mucosal, or unknown primary melanoma treated with >1 infusion of nivolumab, pembrolizumab, or combination ipilimumab/PD-1 inhibitors were included. Response rate, toxicity rate, overall survival (OS), and progression-free survival (PFS) were determined based on infusion timing.

**Results:**

Patients with ≥1 late infusion (after 4 pm) among their first 4 infusions had slightly poorer objective response rate compared with only pre-4 pm infusions (39.7% vs 44.5%), but no significant associations with late infusions and PFS and OS (*P* = .23, .93, respectively). Multivariable analyses showed no statistically significant association with outcomes for patients with any post-4 pm infusion among the first 4; median infusion time was also not associated with outcomes. However, considering all infusion times, we found inferior PFS (median 10.6 vs 38.9 months, *P* < .0001), and numerically inferior OS (median 54.6 vs 81.2 months, *P* = .19) in patients with ≥20% late infusions. Multivariable models had similarly inferior response and PFS for patients with ≥20% late infusions, and later median infusion times were associated with inferior response, PFS, and OS.

**Conclusions:**

Late immunotherapy infusion times were associated with inferior outcomes when considering all infusions, but not when considering initial (first 4) infusions.

## Teaser

Circadian rhythms affect immune function. This retrospective study focused on whether the time of immunotherapy infusion affects outcomes.

**Implications for Practice**

For patients with melanoma, immunotherapy administration times past 4 pm were associated with inferior survival outcomes when considering all infusion times, but not for the initial 4 infusion times. While logistical issues may confound this association, this insight may highlight the importance of circadian rhythm in immunotherapy response and has the potential to maximize existing treatment options.

## Introduction

Immune checkpoint inhibitors have dramatically improved the survival of patients with melanoma. Despite this, some patients either have no response or progress after the initial response. Thus, predictors or effectors of clinical benefit are needed and are the subject of many studies. Potential markers of response range from tumor-specific factors (including genomic or micro-environmental features) to patient factors (including microbiome, body-mass index, and demographic characteristics).^1^

Recently, the impact of circadian rhythm has been studied. Based on preclinical and clinical studies, the human adaptive immune response follows a circadian rhythm, with adaptive immune responses decreasing in evening intervals.^2,3^ Importantly, the adaptive immune response may be a critical component of immunotherapy treatment response.^4^ It was thus hypothesized by Qian et al^5^ that evening immunotherapy treatment times would be correlated with worsened clinical outcomes. In their longitudinal study, patients with melanoma receiving 20% of infusions in the evening were correlated with decreased survival. This finding appeared to be replicated in another small study in head and neck squamous cell carcinoma.^6^ Additional clinical studies have demonstrated an association between morning infusion times and superior outcomes^7-9^; however, a mouse study showed melanoma tumors grew less when anti-PD-1 therapy was administered in the evening.^10^

Thus far, this finding has not been replicated in patients with melanoma, and other infusion timing schemas have not been explored. We speculated that logistical and scheduling issues, in addition to circadian rhythm factors might play a role in this association. In this retrospective study, we assessed clinical outcomes in 516 patients with melanoma relative to the times of day their immunotherapy infusions were administered.

## Materials and methods

### Study design and participants

We performed a longitudinal study in which 516 participants from a single tertiary care center (Vanderbilt University Medical Center, Nashville, TN) diagnosed with cutaneous, acral, mucosal, or unknown primary melanoma were treated with nivolumab, pembrolizumab, or combination ipilimumab/PD-1 inhibitor. Patients were greater than 18 years old at the time of administration. Patients with ocular melanoma were excluded. Patients who received only one infusion due to rapid progression or severe toxicity were also excluded. IRB approval was obtained for the study.

### Data collection

The electronic medical record was reviewed and all infusion times, treatment indication (for adjuvant vs metastatic disease), treatment type (monotherapy vs combination therapy), sex, age, and disease characteristics (stage, performance status, and lactate dehydrogenase) were collected. Treatment start times were recorded by infusion personnel per standard institutional protocols. Treatment outcomes, including best response to treatment, dates of progression, last follow-up, and/or death, and worst-grade immune-related adverse event (irAE) were collected. Response and progression were assessed based on RECIST 1.1; irAE grading was based on CTCAE version 5.03.

Patients were defined as being in the “early group” if they had >80% of their infusions before 4 pm. Patients were defined in the “late group” if they had ≥20% of their infusions after 4 pm (which by definition consisted of ≥1 of the first 4 infusions, or ≥20% of all infusions). To evaluate different cutoffs, we assessed whether all infusions were given before noon versus after noon versus both. We focused on the first 4 infusions since, in most regimens, this comprises all treatments leading up to initial response evaluation (generally given every 3 weeks for 4 treatments followed by cross-sectional imaging), and all ipilimumab/nivolumab combination infusions.

### Statistical analysis

Patient and treatment characteristics were summarized using descriptive statistics. Univariable analyses of the association between infusion time of day and study outcomes were performed using chi-square tests (response and worst-grade toxicity) and log-rank tests (progression-free survival [PFS] and OS). Both nominal and FDR-adjusted *P*-values were reported. Multivariable logistic regression models were used to assess the associations between infusion time of day and worst-grade toxicity. A subgroup analysis was performed among patients with metastatic disease (*n* = 356) for objective response rate (ORR). Cox regression models were used to assess the impact of infusion time on PFS and OS. Models were adjusted for covariates including metastatic stage, histology, elevated LDH, ECOG performance status, monotherapy versus combination therapy, and adjuvant versus metastatic setting (toxicity and survival outcomes only). Adjusted ORs/HRs and 95% CIs are reported. All statistical analyses were performed in R version 4.2.2.

## Results

### Descriptive analysis

A total of 516 patients (187 female [36.2%]; mean [SD] age, 61.1 [14.6] years) were included. Three hundred and seventy (71.7%) patients received anti-PD-1 monotherapy and 146 (28.3%) patients received combination ipilimumab and anti-PD-1. One hundred and sixty patients received adjuvant therapy, while 356 received therapy for metastatic disease. One hundred and forty-eight (28.7%) patients had *BRAF*^V600E^ mutations, and 200 (38.8%) patients had elevated LDH at treatment start; 155 (30.0%) patients were stage I-III, 170 (32.9%) were Stage IV M1a-M1b, and 190 (36.8%) were Stage IV M1c-M1d. Cutaneous melanoma was present in 397 (76.9%), 28 (5.4%) had acral melanoma, 31 (6.0%) had mucosal melanoma, and 60 (11.6%) had melanoma of unknown primary (Table 1).

**Table 1.** Patient characteristics.

	Day only (*N* = 318)	Day andnight (*N* = 198)	Overall (*N* = 516)	*P*-value^a^
Mono vs combo therapy				
Mono	233 (73.3%)	137 (69.2%)	370 (71.7%)	.368
Combo	85 (26.7%)	61 (30.8%)	146 (28.3%)	
Adjuvant vs metastatic				
Adjuvant	101 (31.8%)	59 (29.8%)	160 (31.0%)	.693
Metastatic	216 (67.9%)	139 (70.2%)	355 (68.8%)	
Missing	1 (0.3%)	0 (0%)	1 (0.2%)	
Age				
Mean (SD)	62.2 (14.3)	59.5 (14.9)	61.1 (14.6)	.047
Median [Q1, Q3]	64.0 [54.0, 72.0]	60.5 [49.0, 71.0]	63.0 [51.0, 72.0]	
Missing	1 (0.3%)	0 (0%)	1 (0.2%)	
Stage				
I-III	92 (28.9%)	63 (31.8%)	155 (30.0%)	.573
IV M1a-M1b	110 (34.6%)	60 (30.3%)	170 (32.9%)	
IV M1c-M1d	115 (36.2%)	75 (37.9%)	190 (36.8%)	
Missing	1 (0.3%)	0 (0%)	1 (0.2%)	
Gender				
Female	112 (35.2%)	75 (37.9%)	187 (36.2%)	.624
Male	205 (64.5%)	123 (62.1%)	328 (63.6%)	
Missing	1 (0.3%)	0 (0%)	1 (0.2%)	
Histology				
Cutaneous	247 (77.7%)	150 (75.8%)	397 (76.9%)	.095
Mucosal	15 (4.7%)	16 (8.1%)	31 (6.0%)	
Acral	22 (6.9%)	6 (3.0%)	28 (5.4%)	
Unknown primary	34 (10.7%)	26 (13.1%)	60 (11.6%)	
LDL elevated				
No	191 (60.1%)	111 (56.1%)	302 (58.5%)	.277
Yes	116 (36.5%)	84 (42.4%)	200 (38.8%)	
Missing	11 (3.5%)	3 (1.5%)	14 (2.7%)	
Mutation				
Nonmutated	158 (49.7%)	99 (50.0%)	257 (49.8%)	.802
BRAF V600E	89 (28.0%)	59 (29.8%)	148 (28.7%)	
BRAF V600K	18 (5.7%)	13 (6.6%)	31 (6.0%)	
NRAS	51 (16.0%)	26 (13.1%)	77 (14.9%)	
Missing	2 (0.6%)	1 (0.5%)	3 (0.6%)	
ECOG PS				
0	154 (48.4%)	87 (43.9%)	241 (46.7%)	.349
1+	163 (51.3%)	111 (56.1%)	274 (53.1%)	
Missing	1 (0.3%)	0 (0%)	1 (0.2%)	

^a^These are nominal *P*-values and are not adjusted for multiple comparisons. Wilcoxon Rank Sum tests and chi-Square tests are used for continuous and categorical variables, respectively.

Median PFS (months) and OS in the monotherapy group were 39.0 and 59.1, respectively (among patients with metastatic disease, median PFS and OS were 11.2 and 35.0 months, respectively). The median PFS for the combination therapy group was 20.5 months; the median OS was not reached. Among 356 patients with evaluable responses, 37.7% of patients in the monotherapy group responded, compared with 51.7% of combination therapy patients.

Since cross-sectional imaging is performed after 12 weeks in most patients, we first assessed the initial 4 infusions as those that could impact the initial response to immune therapy. Therefore, we initially focused on the time of day for the initial 4 infusions Supplementary Figure S1 describes the number of patients receiving infusion numbers 1-4 during each hour and their correlating response, toxicity, PFS, and OS.

We assessed whether patients with ≥20% infusions received later in the day correlated had inferior outcomes, as previously described.^5^ Among patients with evaluable responses, there was no difference in ORR (44.5% vs 39.7%, *P* = .19), PFS (median 30.7 vs 22.6 months *P* = .23), or OS (median 59.1 vs 60.0 months *P* = .93) for the first 4 infusions. However, when we considered all infusions (rather than just the first 4), trends seemed to favor the early group (>80% of infusions before 4 pm); ORR was 46.9% vs 31.9% (*P* = .001), median PFS was 38.9 vs 10.6 months (*P* = .0001), and median OS was 81.2 vs 54.6 months (*P* = .19) in the early vs the late groups. High-grade toxicity rates were similar in both groups. Assessing a different cutoff time (noon), we observed that PFS was improved for morning-only infusions for the first 4 and all infusions, and improved OS for all infusions (with a trend to improved for first 4 as well; Supplementary Figure S2).

**Figure 1.**
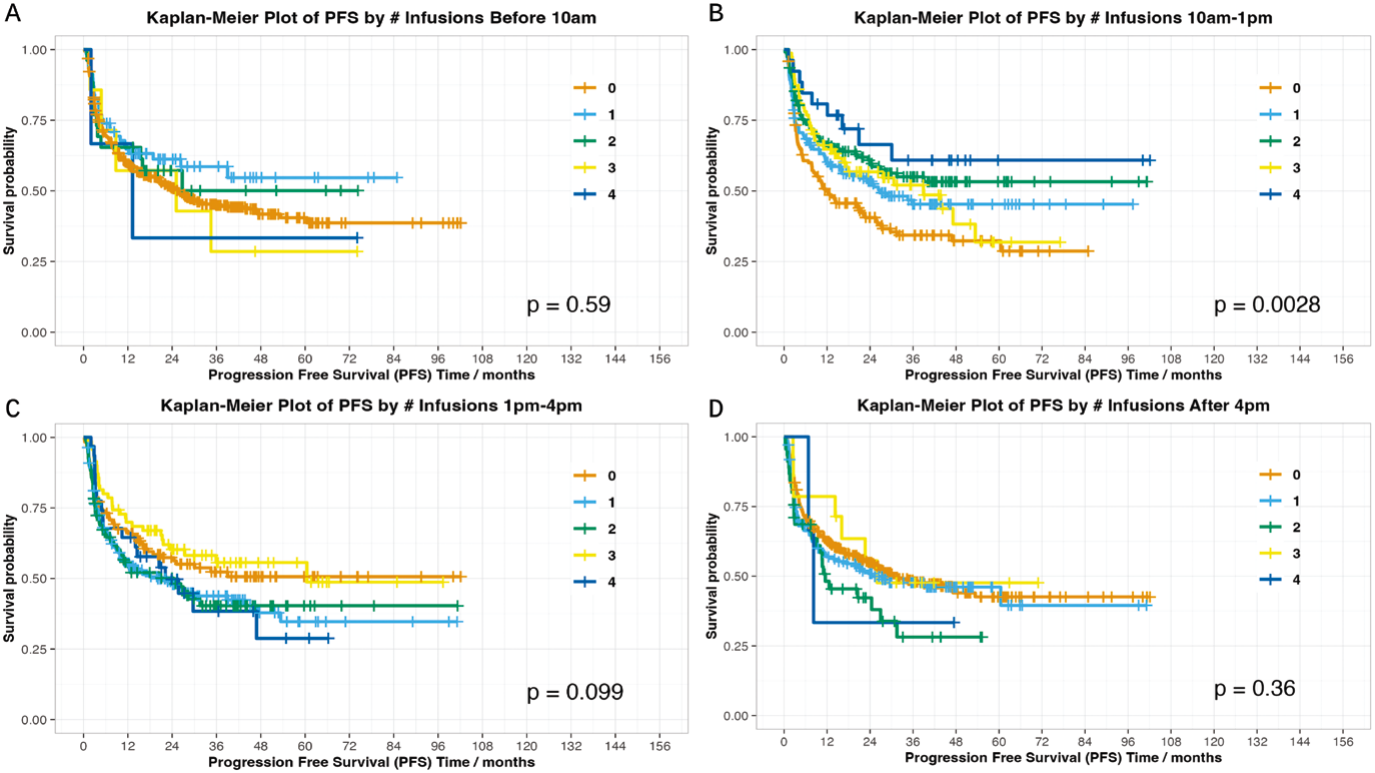
PFS By time frame.

**Figure 2.**
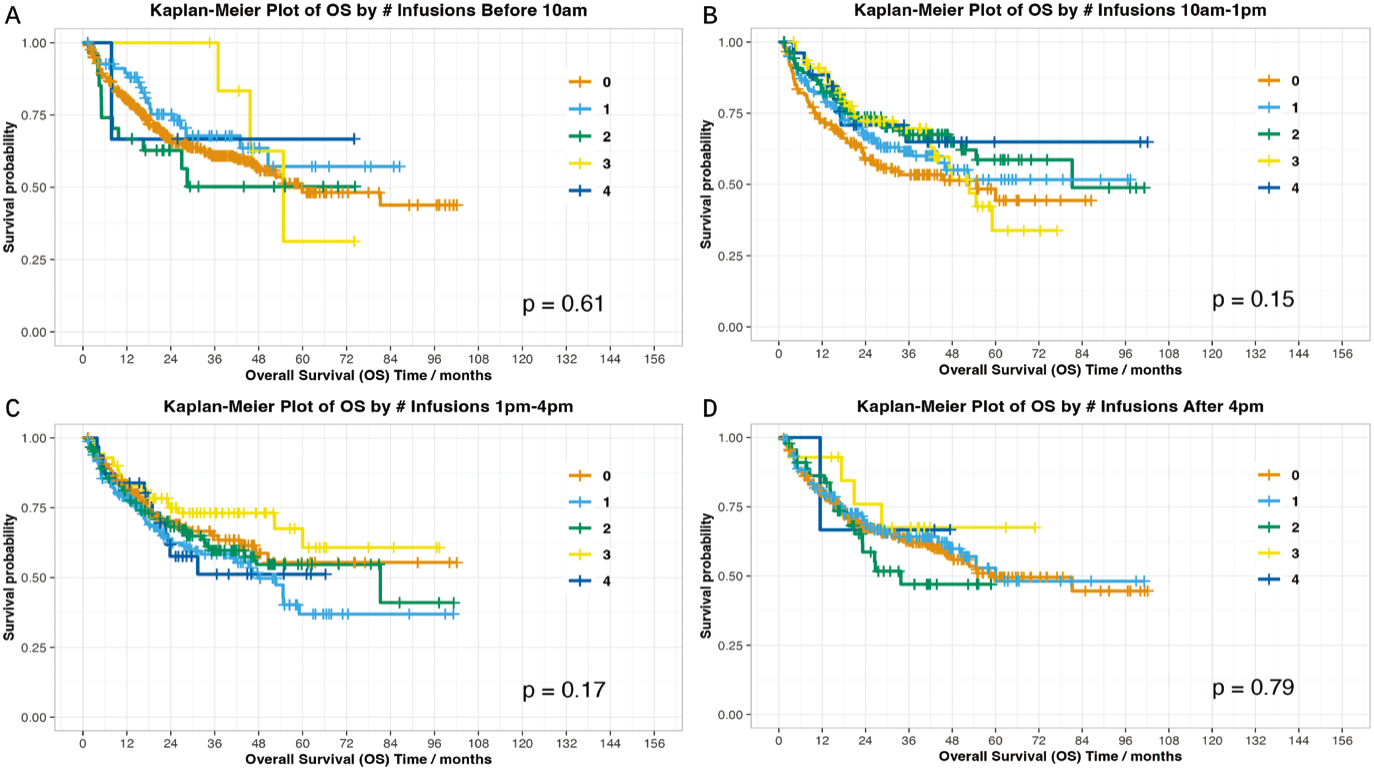
OS by time frame.

To provide further assessment, we assessed whether increasing numbers of infusions at a particular timepoint correlated with clinical outcomes at the patient level (Figures 1, 2). The only statistically significant association was infusions between 10 am and 1 pm and PFS (*P* = .003); no other associations with response, PFS, OS, or worst-grade toxicity were observed. Similar results were seen when comparing patients with 0-1 infusion versus 2-4 infusions at a given time interval, with significant results identified with 2-4 infusions in the 10 am-1 pm timeframe (PFS *P* = .001, OS *P* = .037).

We then performed an exploratory analysis at an infusion level (rather than the patient level) for the first 4 infusions. Among all early infusions (before 4 pm) in patients with evaluable responses, 576/1253 were given to patients with PR/CR (46.0%) compared with 89/221 of infusions given after 4 pm (40.3%; *P* = .12). Similar rates of severe toxicity were observed at the infusion level for the first 4 infusions (14.2% vs 11.1% grade 3-5 toxicities). When grouped by time at the infusion level (before 10 am, 10 am-1 pm, 1 pm-4 pm, and after 4 pm), broadly similar ORR were observed (44.8%, 46.7%, 45.5%, 40.3%, respectively). ORR, PFS, and OS values by time frame for the first 4 infusions at the infusion level are shown in Supplementary Table S1. In contrast, improved response rates were observed when considering all infusions rather than the first 4 (58.7% of pre-4 pm infusions given to responders vs 51.7% after 4 pm, *P* < .01); ORR for before 10 am, 10 am-1 pm, 1 pm-4 pm, and after 4 pm infusions were 62.7%, 60.7%, 55.4%, 51.7%, (*P* < .01). Median PFS, OS, and ORR were also examined for all infusion times per subject (Supplementary Table S2).

### Multivariable analysis

**Figure 3.**
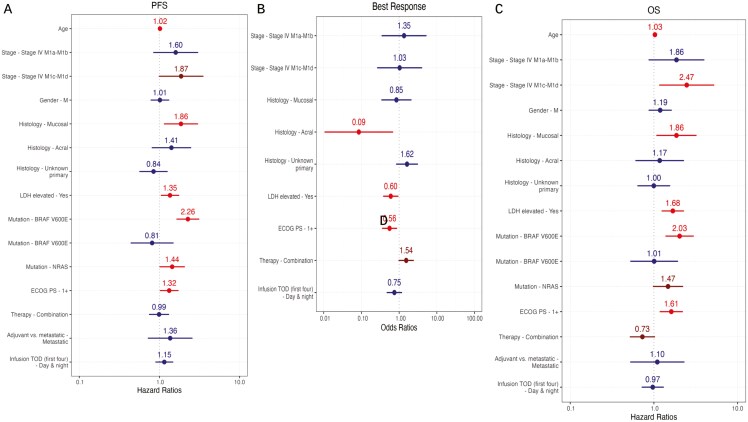
Forest plots showing the impact of at least 1 infusion after 4 pm during the first 4 infusions (“infusion tod first4=day&night”) controlled for age, stage, gender, histology, LDH (lactate dehydrogenase), mutation, ECOG performance status, therapy type, and setting for (A) PFS, (B) Best response, and (C) OS.

We then performed multivariable analyses adjusted for metastatic stage, histology, elevated LDH, ECOG performance status, monotherapy versus combination therapy, and adjuvant versus metastatic setting for the first 4 infusions. Median infusion time was not associated with any clinical outcome. There was also no statistically significant association with OS (*P* = .856), PFS (*P* = .315), toxicity (*P* = .1), or ORR (*P* = .219) for patients with any post-4 pm infusion among the first 4. Forest plots for the first 4 infusion times can be seen in Figure 3. Results were similar when assessing only therapy in the metastatic setting, and only cutaneous melanoma or unknown primary, with no significant association between median infusion time or any post-4 pm infusion time. Results were overall similar using a noon cutoff, with significant outcomes when considering all infusions but less so when considering the first 4 (Supplementary Figures S3, S4).

We then assessed the number of infusions within the predefined time frames (before 10 am, 10 am-1 pm, 1 pm-4 pm, and after 4 pm) for the first 4 infusion times. Patients receiving infusions between 10 am and 1 pm had better ORR (OR 1.28, 95% CI 1.05-1.56, *P* = .014) as well as improved PFS (HR 0.85, 95% CI 0.76-0.94, *P* = .003) but not OS (*P* = .079) or toxicity (*P* = .189). Notably, receiving more infusions after 4 pm was significantly associated with lower rates of severe toxicity (OR 0.58, 95% CI 0.39-0.87, *P* = .008). There was no significant association between any other time frames and response, OS, and PFS.

**Figure 4.**
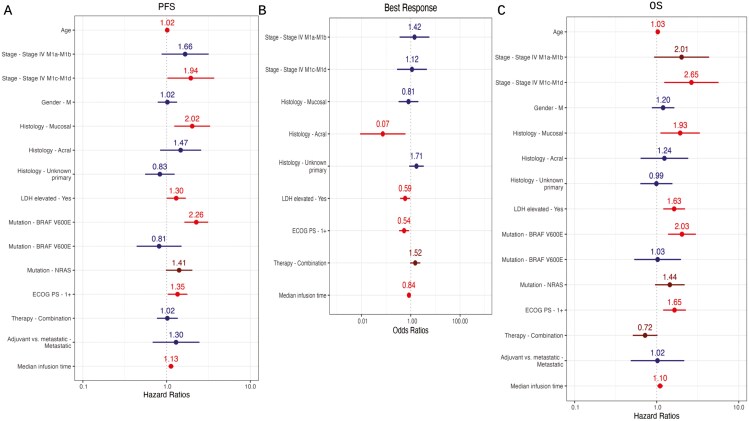
Forest plots showing the impact of median infusion time (“med infusion all”) during all anti-PD-1 infusions controlled for age, stage, gender, histology, LDH (lactate dehydrogenase), mutation, ECOG performance status, therapy type, and setting for (A) PFS, (B) Best response, and (C) OS.

**Figure 5.**
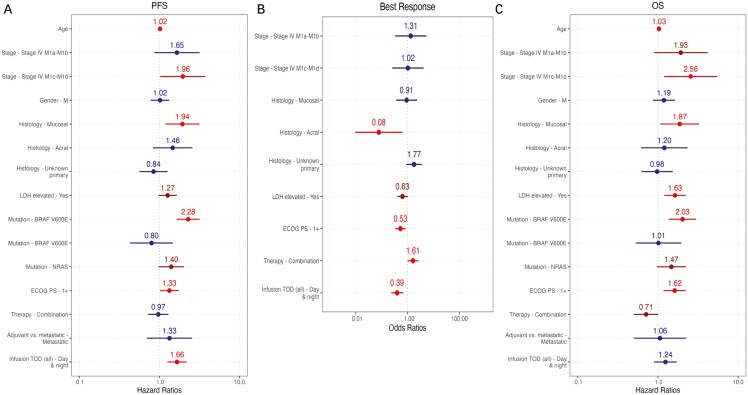
Forest plots showing the impact of >20% of infusions after 4 pm during all anti-PD-1 infusions (“infusion tod all=day&night”) controlled for age, stage, gender, histology, LDH (lactate dehydrogenase), mutation, ECOG performance status, therapy type, and setting for (A) PFS, (B) Best response, and (C) OS.

We then performed multivariable analyses for all infusions (as opposed to the first 4) with the same adjusted variables as above. Notably, patients with >20% of their infusions after 4 pm had inferior odds of response (OR 0.39, *P* = .001) and inferior PFS (HR 1.66, *P* < .001), with similar OS (HR 1.24, *P* = .20) and similar rate of toxicities (OR 0.9, *P* = .76; Figure 4). Similarly, later median infusion times were associated with inferior odds of response (0.84, *P* = .008), inferior PFS (HR 1.13, *P* = .001), inferior OS (HR 1.10, *P* = .022), and similar rates of toxicity (OR 1.02, *P* = .84; Figure 5). Results were similar for considering only patients with metastatic disease and cutaneous/unknown primary melanoma (Supplementary Figure S5).

Given the discordance between infusion time and outcomes between the first 4 infusions and all infusions, we speculated that patients who are benefiting from therapy and receiving longer courses of therapy may have a propensity to have earlier infusions due to scheduling preferences, whereas initial treatments may be scheduled later in the day as overbook appointments, or more urgent scheduling. Accordingly, median treatment time for months 0-3, 3-6, 6-12, and beyond 12 months were 1:18 pm, 12:30 pm, 12:30 pm, and 12:48 pm.

## Discussion

In this retrospective study of 516 patients at a single treatment center, anti-PD-1-treated patients who received more than 20% of infusions after 4 pm had worse PFS, and OS, although these associations were not present when considering only the first 4 infusions (ie, those that contributed to the initial response evaluation). The discordance between considering the initial versus all infusions is not clear and could be related to biological differences related to circadian rhythm, or could be related to logistical issues (eg, patients who receive longer courses of therapy may schedule earlier infusion times, as borne out by data suggesting that median treatment time was earlier for patients with more extended treatment courses).

Previous research from Qian et al suggested that evening immunotherapy infusion times correlate with worse outcomes. This study combined with Qian et al could support the impact of circadian rhythm on immunotherapy and the immune system as a whole. Recent studies demonstrate that lymphocytes and macrophages have their own internal clocks.^11-14^ Some of our data support these studies, including the decreasing response rate (at an infusion level) with later time of infusion. Counterbalancing this consideration could be the long half-life of ICI agents, thus questioning the biological plausibility of whether individual infusion times could impact outcomes. Randomized studies and studies in other cancers could help validate these associations.

However, we must also consider the possibility that these results arrived by chance alone, or because of logistical issues. One could posit that early infusions may have more time variance due to urgent scheduling and overbooking, while later infusions are scheduled further in advance, potentially resulting in earlier appointment times. With this scenario, patients who respond/benefit from treatment may have similar time distribution initially but have earlier infusion times later in their treatment course (eg, we observed that the median infusion time was 1:18 pm for the first 3 months, but 12:30 pm for months 3-12). Thus, responding patients by definition would have earlier median infusion times and less likelihood of later infusion times. In addition, many statistical comparisons were completed in this study and could result in significant results by chance alone. Additionally, toxicities were only marginally associated with infusion times (fewer toxicities in patients with late infusion times in the first 4 infusions).

### Limitations

Limitations of the study included its retrospective and single-center nature. Toxicity reporting may lack the accuracy of a formal clinical trial. There may be demographic and socioeconomic variables influencing outcomes, such as annual income or sleep schedule, that we are unable to account for here due to limitations in data availability. In addition, the factors that lead to different infusion time preferences are not clear and may introduce unquantifiable biases into the analysis.

## Conclusions

Infusion times past 4 pm were associated with inferior outcomes when considering all infusions, but not for initial infusion times. Insights into the optimization of immunotherapy are crucial to maximize existing treatment options, although logistical issues could also confound this association.

## Supplementary Material

Supplementary material is available at *The Oncologist* online.

oyae197_suppl_Supplementary_Figures_1-5_Tables_1-2

## Data Availability

The data underlying this article will be shared on reasonable request to the corresponding author.
