# Supplementary material for: Immune checkpoint inhibitor infusion times and clinical outcomes in patients with melanoma
Source: Oncologist. 2024 Aug 27;30(1):oyae197. doi: 10.1093/oncolo/oyae197 (PMC11783311; doi:10.1093/oncolo/oyae197)
Supplement: oyae197_suppl_Supplementary_Figures_1-5_Tables_1-2 [file oyae197_suppl_supplementary_figures_1-5_tables_1-2.zip › Supplementary Figure Captions.pdf]

Supplementary Figure Captions

Supplementary Figure 1: Clock Plot of First Four Infusion Times for a) best response b) worst grade toxicity c) PFS status and d) OS status

Supplementary Figure 2: A) PFS and B) OS comparing patients who had (1) all of their first four infusions in morning (before 12pm), (2) all of their first four infusions in the afternoon/evening, or (3) a combination of both morning and afternoon/evening.; C) PFS and D) OS comparing (1) $\geqslant$ 80% of their first four infusions in morning (before 12pm), (2) all of their first four infusions in the afternoon/evening, or (3) a combination of both morning and afternoon/evening.

Supplementary Figure 3: We redefined the infusion TOD cut point to 12pm. Patients could have infusions in the “Morning only”, “Afternoon/evening only”, or “Morning & afternoon/evening” for the first four infusions. A) PFS and B) OS

Supplementary Figure 4: We redefined the infusion TOD cut point to 12pm. Patients could have infusions in the “Morning only”, “Afternoon/evening only”, or “Morning & afternoon/evening” for all infusions. A) PFS and B) OS

Supplementary Figure 5: Forest Plots for A) PFS, B) Best response, C) OS, and D) Worst grade toxicity including only patients with metastatic therapies and cutaneous/unknown primary histology
