# Supplementary material for: Immune checkpoint inhibitor infusion times and clinical outcomes in patients with melanoma
Source: Oncologist. 2024 Aug 27;30(1):oyae197. doi: 10.1093/oncolo/oyae197 (PMC11783311; doi:10.1093/oncolo/oyae197)
Supplement: oyae197_suppl_Supplementary_Figures_1-5_Tables_1-2 [file oyae197_suppl_supplementary_figures_1-5_tables_1-2.zip › rev Supp Table 2-The Oncologist.docx]

Supplementary Table 2: Infusion Level Outcomes for All Infusion Times

|  | **Before 10am (N=715)** | **10am - 1pm (N=2516)** | **1pm - 4pm (N=2266)** | **After 4pm (N=792)** | **Overall (N=6289)** |
| --- | --- | --- | --- | --- | --- |
| **Best response*** |  |  |  |  |  |
| SD/PD | 208 (32.3%) | 765 (39.3%) | 837 (44.6%) | 315 (48.3%) | 2125 (42.2%) |
| CR/PR | 350 (62.7%) | 1184 (60.7%) | 1040 (55.4%) | 337 (51.7%) | 2911 (57.8%) |
| **Worst grade toxicity** |  |  |  |  |  |
| 1-2 | 359 (50.2%) | 1342 (53.3%) | 1117 (49.3%) | 419 (52.9%) | 3237 (51.5%) |
| 3-5 | 102 (14.3%) | 245 (9.7%) | 254 (11.2%) | 77 (9.7%) | 678 (10.8%) |
| **PFS Status** |  |  |  |  |  |
| 0 | 455 (63.6%) | 1486 (59.1%) | 1173 (51.8%) | 402 (50.8%) | 3516 (55.9%) |
| 1 | 260 (36.4%) | 1028 (40.9%) | 1086 (47.9%) | 388 (49.0%) | 2762 (43.9%) |
| **PFS (months)** |  |  |  |  |  |
| Median | Not reached | 60.5 | 43.7 | 38.9 |  |
| **OS Status** |  |  |  |  |  |
| 0 | 573 (80.1%) | 1792 (71.2%) | 1534 (67.7%) | 552 (69.7%) | 4451 (70.8%) |
| 1 | 142 (19.9%) | 722 (28.7%) | 725 (32.0%) | 238 (30.1%) | 1827 (29.1%) |
| **OS (months)** |  |  |  |  |  |
| Median | Not reached | Not reached | 81.2 | Not reached |  |

*Note: Percentages listed for Best Response reflect only evaluable responses and thus their N differs from the N in the heading.
