# Supplementary material for: Immune checkpoint inhibitor infusion times and clinical outcomes in patients with melanoma
Source: Oncologist. 2024 Aug 27;30(1):oyae197. doi: 10.1093/oncolo/oyae197 (PMC11783311; doi:10.1093/oncolo/oyae197)
Supplement: oyae197_suppl_Supplementary_Figures_1-5_Tables_1-2 [file oyae197_suppl_supplementary_figures_1-5_tables_1-2.zip › rev Suppl Table 1- The Oncologist.docx]

Supplementary Table 1: Infusion Level Outcomes by Time Group for First Four Infusions

|  | **Before 10am (N=156)** | **10am - 1pm (N=727)** | **1pm - 4pm (N=742)** | **After 4pm (N=280)** | **Overall (N=1905)** |
| --- | --- | --- | --- | --- | --- |
| **Best response*** |  |  |  |  |  |
| SD/PD | 69 (55.2%) | 285 (53.3%) | 323 (54.5%) | 132 (59.7%) | 809 (54.9%) |
| CR/PR | 56 (44.8%) | 250 (46.7%) | 270 (45.5%) | 89 (40.3%) | 665 (45.1%) |
| **Worst grade toxicity** |  |  |  |  |  |
| 1-2 | 71 (45.5%) | 331 (45.5%) | 307 (41.4%) | 142 (50.7%) | 851 (44.7%) |
| 3-5 | 29 (18.6%) | 88 (12.1%) | 114 (15.4%) | 31 (11.1%) | 262 (13.8%) |
| **PFS Status** |  |  |  |  |  |
| 0 | 81 (51.9%) | 401 (55.2%) | 355 (47.8%) | 128 (45.7%) | 965 (50.7%) |
| 1 | 75 (48.1%) | 324 (44.6%) | 385 (51.9%) | 152 (54.3%) | 936 (49.1%) |
| **PFS (months)** |  |  |  |  |  |
| Median | 34.5 | 47.1 | 26.9 | 21.6 |  |
| **OS Status** |  |  |  |  |  |
| 0 | 99 (63.5%) | 479 (65.9%) | 466 (62.8%) | 178 (63.6%) | 1222 (64.1%) |
| 1 | 57 (36.5%) | 246 (33.8%) | 274 (36.9%) | 102 (36.4%) | 679 (35.6%) |
| **OS (months)** |  |  |  |  |  |
| Median | 54.8 | 81.2 | 81.2 | Not reached |  |

*Note: Percentages listed for Best Response reflect only evaluable responses and thus their N differs from the N in the heading.
