# Supplementary material for: Immune checkpoint inhibitor infusion times and clinical outcomes in patients with melanoma
Source: Oncologist. 2024 Aug 27;30(1):oyae197. doi: 10.1093/oncolo/oyae197 (PMC11783311; doi:10.1093/oncolo/oyae197)

Supplementary Figure 2

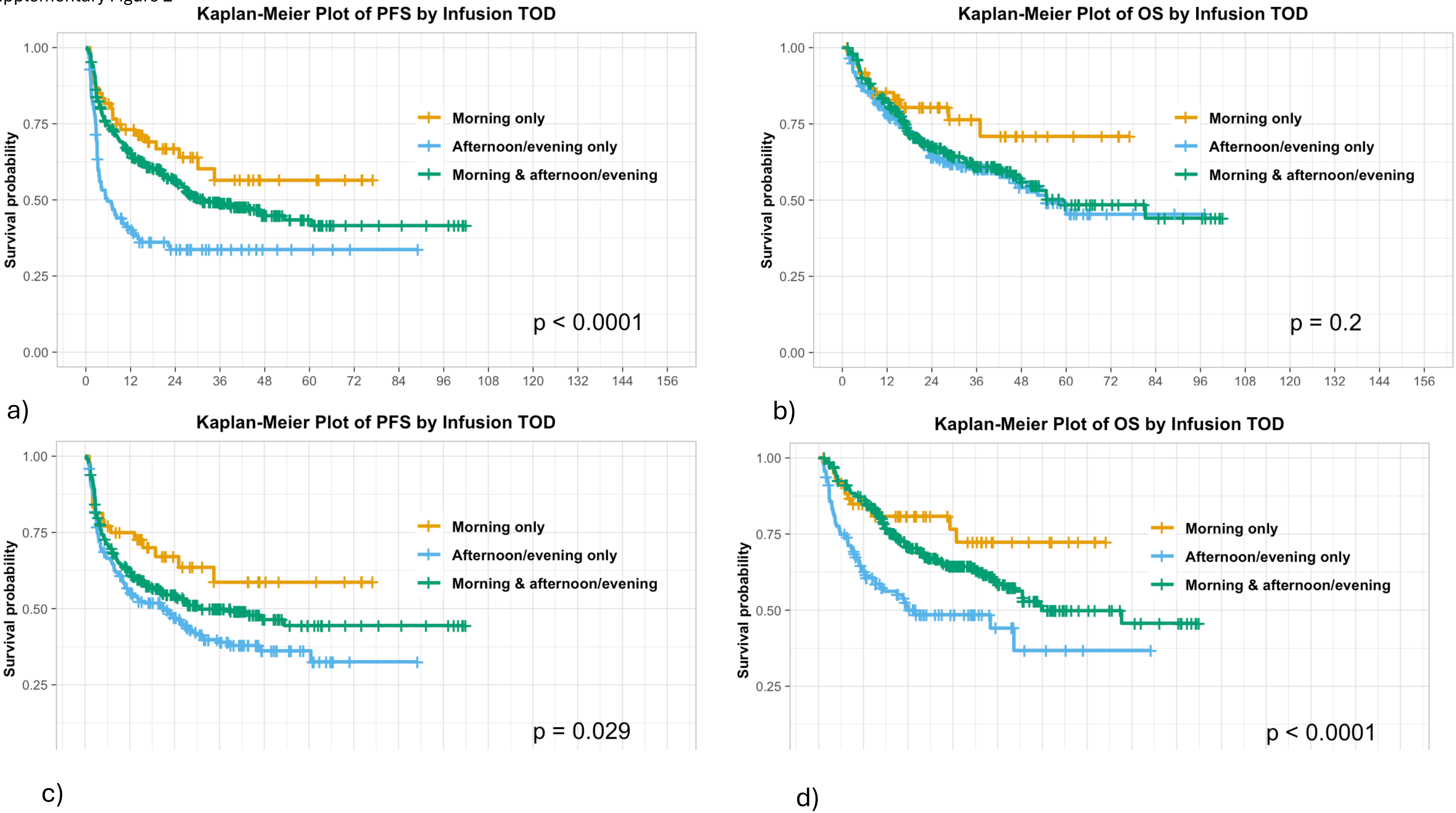

Supplement: oyae197_suppl_Supplementary_Figures_1-5_Tables_1-2 [file oyae197_suppl_supplementary_figures_1-5_tables_1-2.zip › rev_SuppFig2_TOD.pdf]
