# Supplementary figures and images for: Immune checkpoint inhibitor infusion times and clinical outcomes in patients with melanoma
Source: Oncologist. 2024 Aug 27;30(1):oyae197. doi: 10.1093/oncolo/oyae197 (PMC11783311; doi:10.1093/oncolo/oyae197)

Supplementary Figure 5

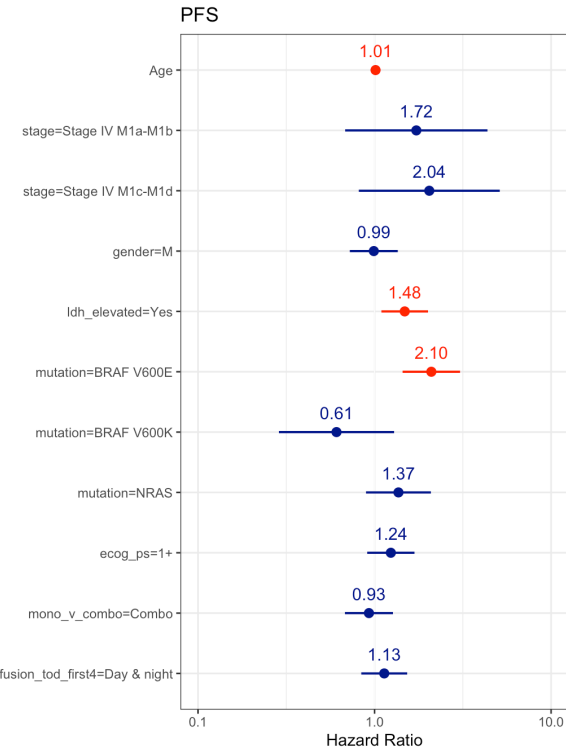

a)

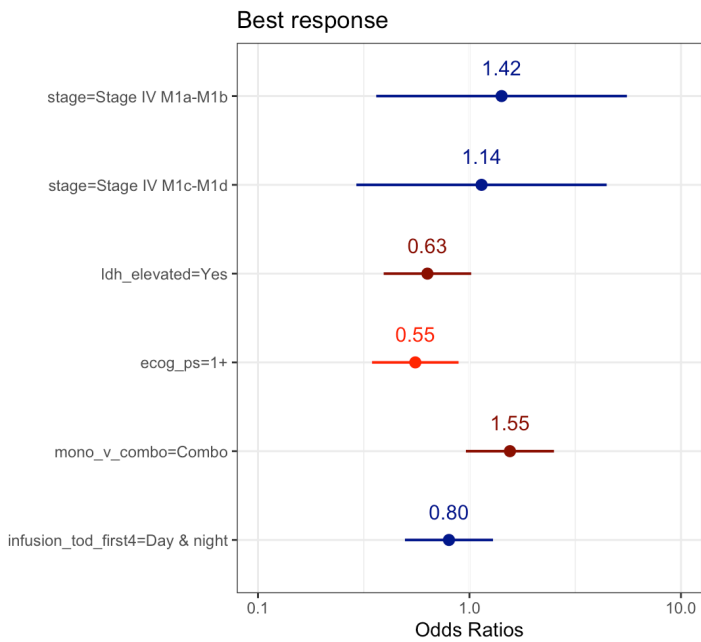

b)

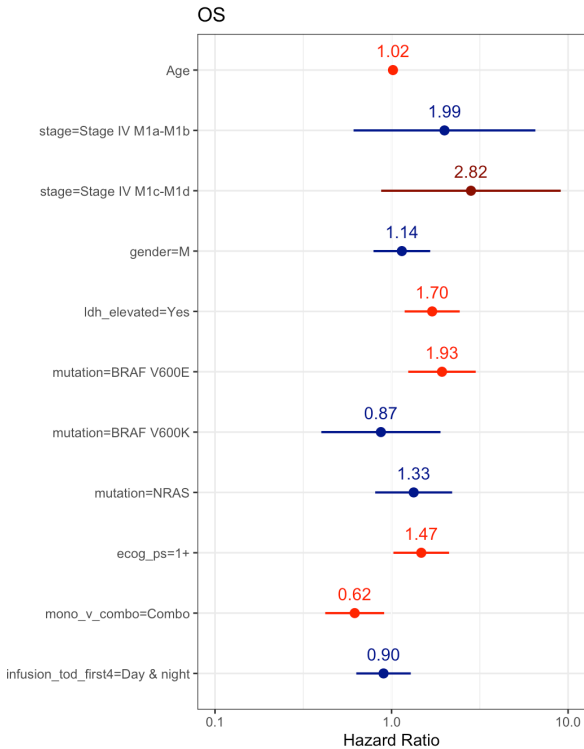

c)

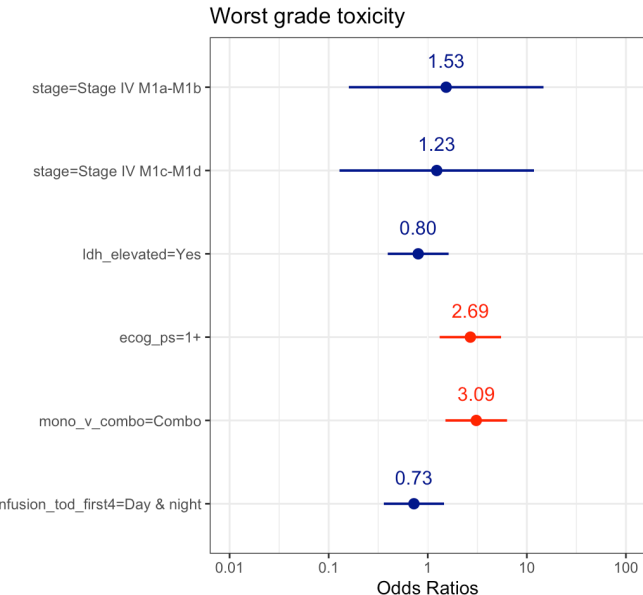

d)

Supplement: oyae197_suppl_Supplementary_Figures_1-5_Tables_1-2 [file oyae197_suppl_supplementary_figures_1-5_tables_1-2.zip › rev_SuppFig5_TOD.pdf]

Supplementary Figure 1

Best Response

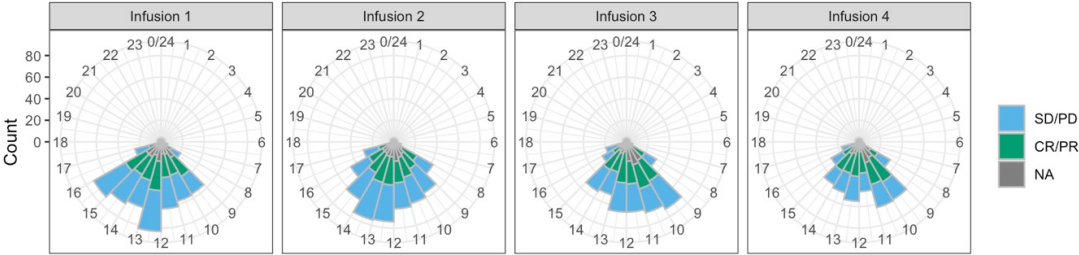

a)

Worst Grade Toxicity

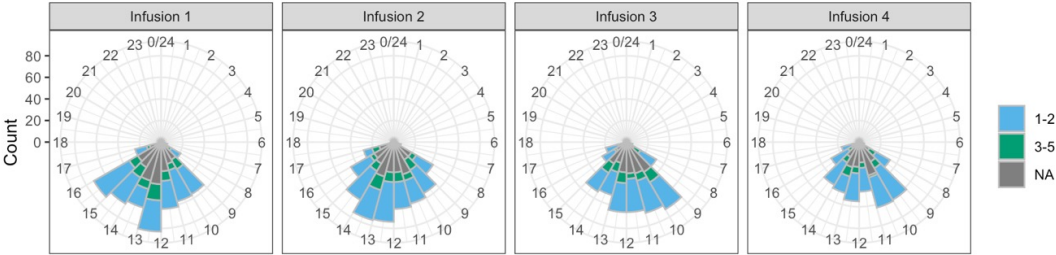

b)

PFS Status

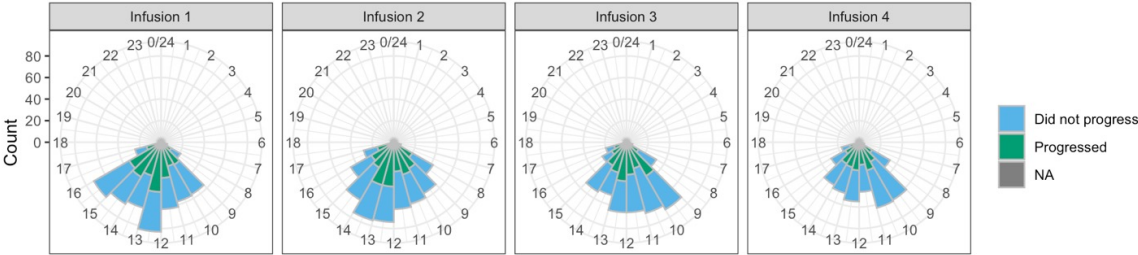

c)

OS Status

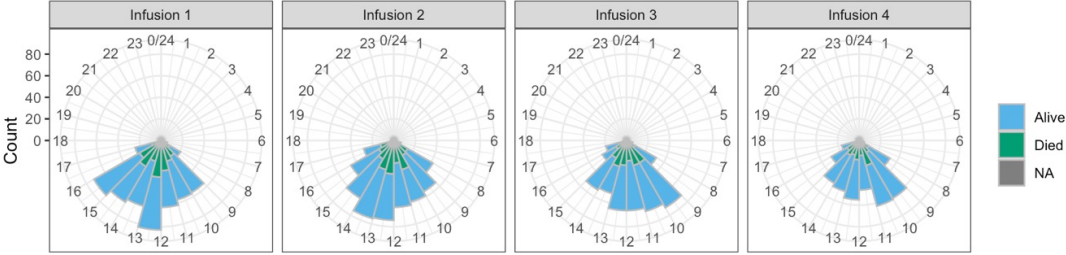

Supplement: oyae197_suppl_Supplementary_Figures_1-5_Tables_1-2 [file oyae197_suppl_supplementary_figures_1-5_tables_1-2.zip › SupFig1.pdf]

Supplementary Figure 3

PFS

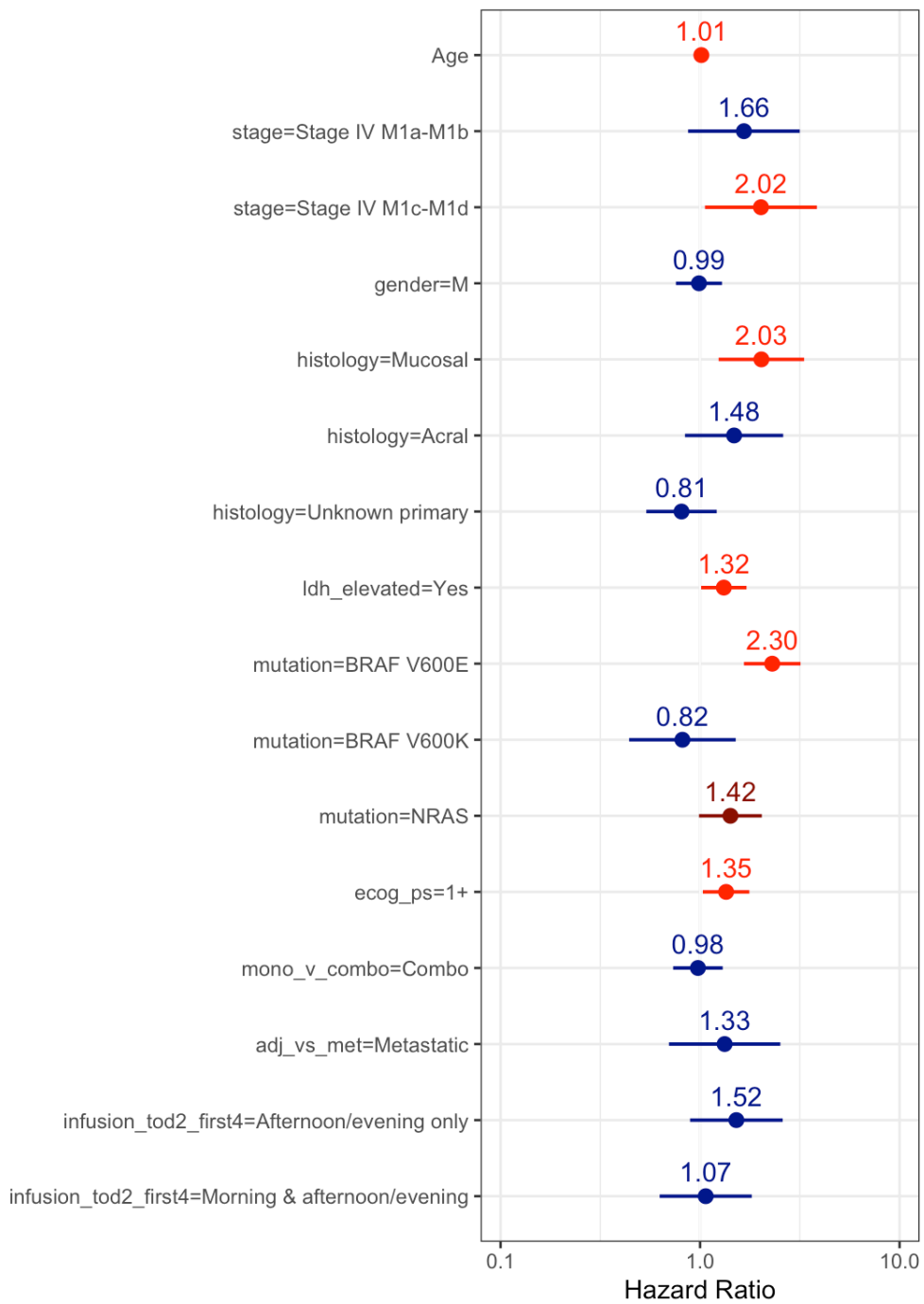

a)

OS

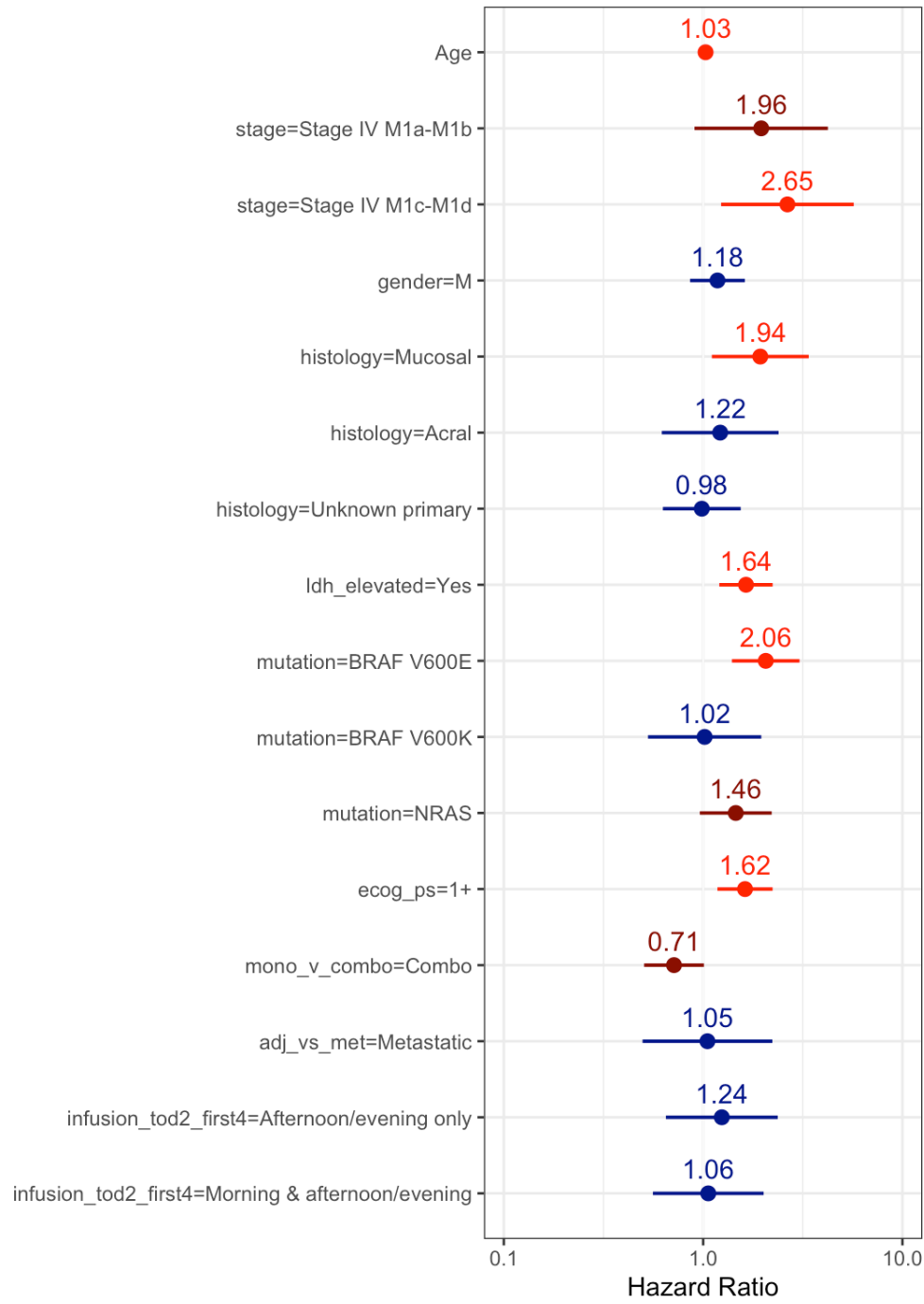

b)

Supplement: oyae197_suppl_Supplementary_Figures_1-5_Tables_1-2 [file oyae197_suppl_supplementary_figures_1-5_tables_1-2.zip › rev_SuppFig3.pdf]

Supplementary Figure 4

PFS

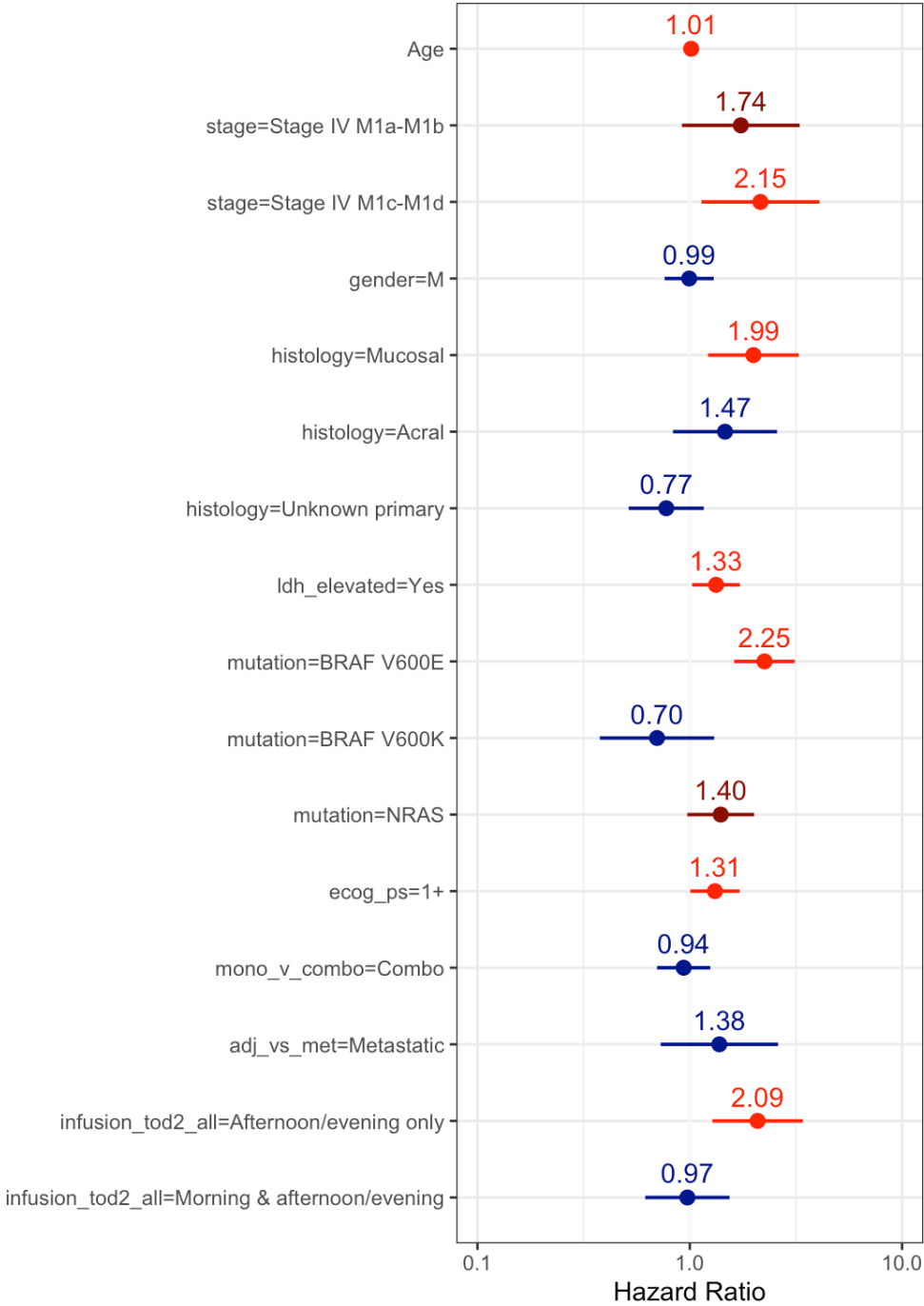

a)

OS

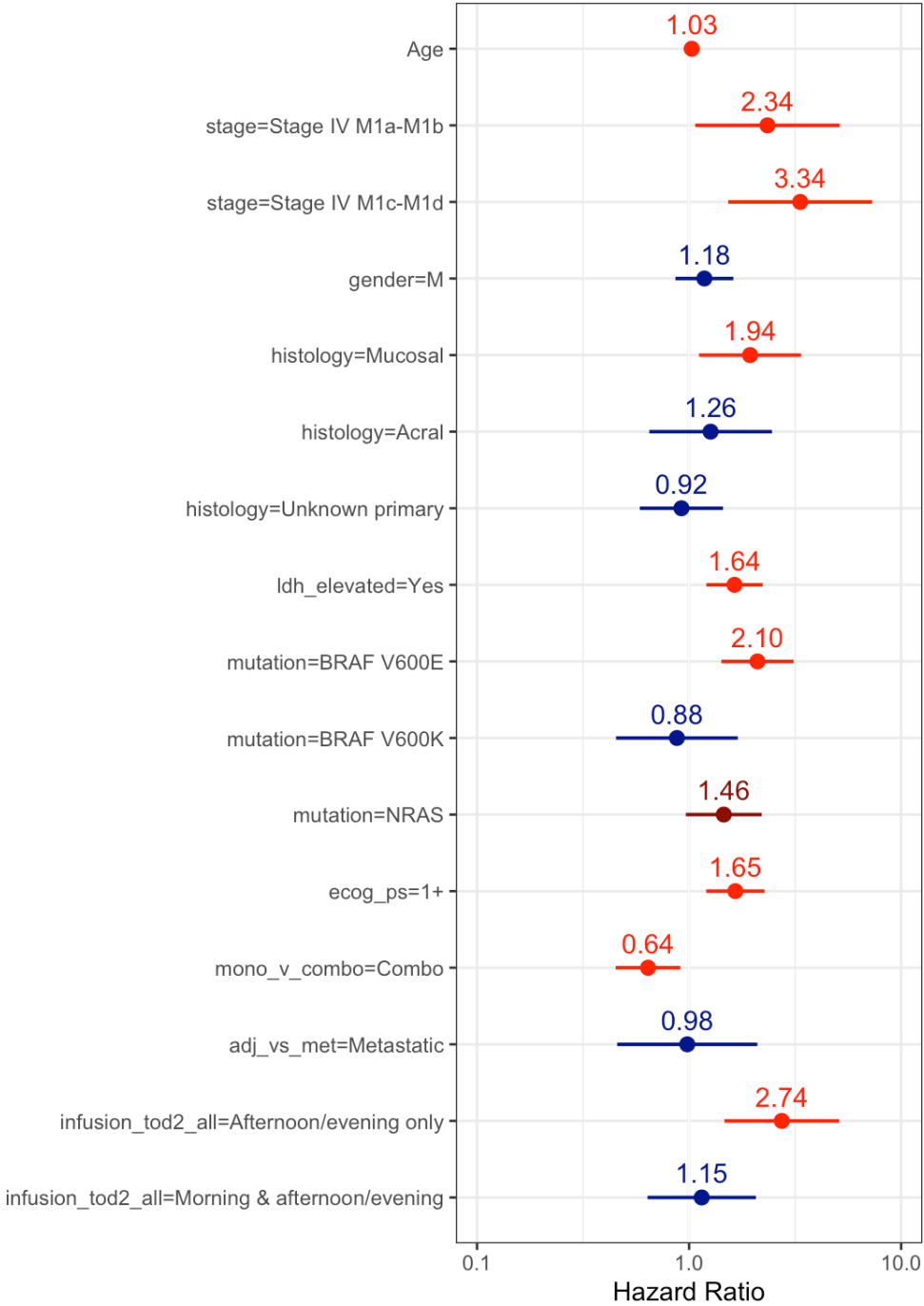

b)

Supplement: oyae197_suppl_Supplementary_Figures_1-5_Tables_1-2 [file oyae197_suppl_supplementary_figures_1-5_tables_1-2.zip › rev_SuppFig4_TOD.pdf]
